# Sepsis-associated brain injury: underlying mechanisms and potential therapeutic strategies for acute and long-term cognitive impairments

**DOI:** 10.1186/s12974-022-02464-4

**Published:** 2022-04-29

**Authors:** Nobufumi Sekino, Magdy Selim, Amjad Shehadah

**Affiliations:** 1 Department of Medicine, Translational Therapeutics Division, Beth Israel Deaconess Medical Center, Harvard Medical School, Boston, MA 02215 USA; 2 Department of Neurology, Stroke and Cerebrovascular Diseases Division, Beth Israel Deaconess Medical Center, Harvard Medical School, 330 Brookline Avenue, CLS-641, Boston, MA 02215 USA

**Keywords:** Sepsis, Cognitive impairment, Neuroinflammation, White matter change, Alzheimer’s disease, Amyloid-beta, Tau protein

## Abstract

Sepsis is a life-threatening organ dysfunction caused by a dysregulated host response to infection. Sepsis causes cerebral dysfunction in the short and long term and induces disruption of the blood–brain barrier (BBB), neuroinflammation, hypoperfusion, and accumulation of amyloid β (Aβ) and tau protein in the brain. White matter changes and brain atrophy can be detected using brain imaging, but unfortunately, there is no specific treatment that directly addresses the underlying mechanisms of cognitive impairments in sepsis. Here, we review the underlying mechanisms of sepsis-associated brain injury, with a focus on BBB dysfunction and Aβ and tau protein accumulation in the brain. We also describe the neurological manifestations and imaging findings of sepsis-associated brain injury, and finally, we propose potential therapeutic strategies for acute and long-term cognitive impairments associated with sepsis. In the acute phase of sepsis, we suggest using antibiotics (such as rifampicin), targeting proinflammatory cytokines, and preventing ischemic injuries and hypoperfusion. In the late phase of sepsis, we suggest targeting neuroinflammation, BBB dysfunction, Aβ and tau protein phosphorylation, glycogen synthase kinase-3 beta (GSK3β), and the receptor for advanced glycation end products (RAGE). These proposed strategies are meant to bring new mechanism-based directions for future basic and clinical research aimed at preventing or ameliorating acute and long-term cognitive impairments in patients with sepsis.

## Introduction

Sepsis is a systemic inflammatory disease defined as a life-threatening organ dysfunction caused by a dysregulated host response to infection [1]. Sepsis is a global health problem affecting 49 million people and causing 11 million deaths each year worldwide [2]. The mortality rate of sepsis has been declining, but it is still remarkably high, ranging between 15 and 25%. In-hospital mortality of patients with septic shock could be as high as 30% to 50% [3]. Studies have shown that many patients with sepsis experience acute and long-term cognitive impairments [4–6]. However, no specific treatment that directly addresses the underlying mechanisms of cognitive impairments in sepsis currently exists, and many controversies surround the optimal treatment approach for these patients. Advances in therapy for sepsis are urgently needed to improve clinical outcomes [7]. Here, we review the underlying mechanisms of sepsis-associated brain injury, with a focus on blood–brain barrier (BBB) dysfunction, neuroinflammation, and amyloid β (Aβ) and tau protein accumulation in the brain. We also describe the neurological manifestations and imaging findings of sepsis-associated brain injury, and finally, we propose potential therapeutic strategies for acute and long-term cognitive impairments associated with sepsis.

## Underlying mechanisms of sepsis-associated brain injury

**Fig. 1:**
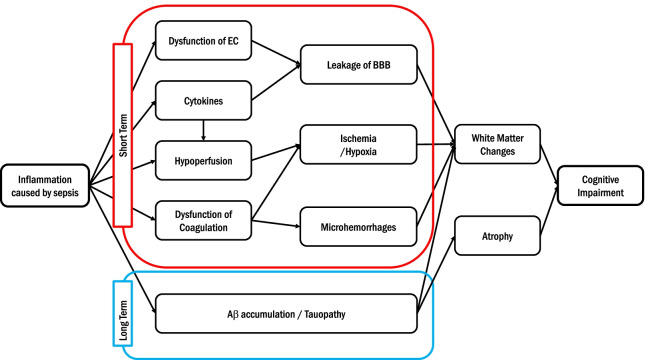
Schematic representation of underlying mechanisms of sepsis-related brain injuries. Inflammation caused by sepsis affects a wide range of processes. In the short term, it induces dysfunction of endothelial cells (EC) leading to blood–brain barrier (BBB) leakage. Proinflammatory cytokines can lead to BBB leakage and contribute to hypoperfusion. Hypoperfusion and dysfunction of coagulation induce ischemia/hypoxia and microhemorrhage. These factors ultimately lead to changes in the white matter in the brain. Sepsis-related inflammation may have a long-term effect. Sepsis may lead to amyloid β (Aβ) and tau protein in the brain. Ultimately, these processes lead to cognitive impairment

Multiple mechanisms contribute to brain damage in sepsis, including systemic inflammation, neuroinflammation, BBB dysfunction, ischemia/hypoxia due to hypoperfusion, Aβ and tau protein accumulation, and direct damage to the brain (Fig. 1 and Table 1). Notably, systemic inflammation in sepsis can exacerbate BBB dysfunction, hypoperfusion, as well as, accumulation of both Aβ and tau protein. On the other hand, BBB disruption induces brain inflammation and may ultimately cause white matter changes and cerebral dysfunction.

**Table 1:** Summary of the main topics discussed in this review

Topic	References
Underlying mechanisms of sepsis	
a. Sepsis modeling in animals	[8–12]
b. From systemic inflammation to neuroinflammation and BBB dysfunction	
- Systemic inflammation	[3, 13–18]
- Neuroinflammation	[19–25, 27–33]
- BBB dysfunction	[34–50]
c. Ischemia/hypoperfusion	[5, 51–57]
d. Aβ and tau protein accumulation	[58–69]
- GSK3β	[70–77]
- RAGE	[60, 63, 78–88]
e. Direct brain damage	[89, 90]
Neurological manifestations of sepsis-associated brain injury	
a. The spectrum of acute cognitive impairments in sepsis	[91, 93, 94]
- Sepsis-associated encephalopathy	[29, 91, 94–96]
- Delirium	[97–100]
- Sickness behavior	[101–104]
b. Long-term cognitive impairments in sepsis	[4–6, 91, 94, 105–107]
Imaging findings in sepsis-associated brain injury	[39, 89, 92, 108–110, 112–117]
Potential therapeutic strategies	
a. Acute phase therapeutic strategies	
- Antibiotics	[5, 118–121]
- Targeting proinflammatory cytokines	[4, 103, 122–125]
- Prevention of hypovolemia and cerebral ischemia	[39, 126–132]
b. Long-term therapeutic strategies	
- Targeting neuroinflammation	[26–28, 133–139]
- Target BBB	[39, 40, 42–44, 140–146]
- Targeting Aβ and tau phosphorylation	[58, 59, 61–63, 147]
- Targeting GSK3β	[70, 71, 148, 149]
- Targeting RAGE	[60, 63, 78, 79, 85, 150–152]

*Aβ* Amyloid β, *BBB* blood–brain barrier, *GSK3β* glycogen synthase kinase-3 beta, *RAGE* receptor for advanced glycation end products

### Sepsis modeling in animals

Although no one model reflects all the clinical complexities of sepsis, animal modeling is a valuable way to study its underlying mechanisms and to develop new therapeutic strategies [8, 9]. The main two animal models used in sepsis research are the cecal ligation and puncture (CLP) and lipopolysaccharide (LPS) models. The CLP model mimics the nature and evolution of severe sepsis in humans [10]. Following a simple procedure to ligate and puncture the cecum, the CLP model induces sepsis as a result of stercoral peritonitis, followed by polymicrobial translocation in the blood circulation [8]. During gram-negative bacterial infection and other acute illnesses, LPS is a major mediator in sepsis [11]. In the LPS model, endotoxins are infused intravascularly, which results in an early and transient peak of cytokines, whereas in the CLP model, the pro-inflammatory response is delayed and persists over a longer period [9]. Due to the effects of the intense inflammatory response on the cardiovascular system, mortality in the LPS model occurs early, whereas in the CLP model, the mortality is delayed and occurs due to multiple organ failure as a complication of the induced peritonitis [8, 12].

### From systemic inflammation to neuroinflammation and BBB dysfunction

#### Systemic inflammation

Sepsis is fundamentally a systemic inflammatory disease mediated by the activation of the innate immune system [3]. The activation of the innate immune response is mediated by pattern recognition receptors (PRRs), such as Toll-like receptors (TLRs), nucleotide-binding oligomerization domain (NOD)-like receptors, retinoic acid-inducible gene (RIG)-like receptors, mannose-binding lectin, and scavenger receptors [3, 13]. PRRs detect both pathogen-associated molecular patterns (PAMPs) and damage-associated molecular patterns (DAMPs), which initiate the upregulation of inflammatory-related genes [14] and induce a complex intracellular signaling system [15]. The transcription complex nuclear factor kappa B (NF-κB) is triggered in response to numerous extracellular inflammatory stimuli [16]. The activation of NF-κB induces the expression of early activation genes, including tumor necrosis factor alpha (TNF-α), interleukin (IL)‑1, IL‑12, IL‑18, and type I interferons (IFNs), among others. These cytokines initiate a cascade of other inflammatory cytokines and chemokines, including IL‑6, IL‑8, IFNγ, CC–chemokine ligand 2 (CCL2), CCL3, and CXC–chemokine ligand 10 (CXCL10), exacerbating the inflammatory response [3, 17]. The activation of these inflammatory networks begins within minutes of PAMP or DAMP recognition [3]. Maciel et al. reported that plasma levels of IL-6 and IL-10 in patients at hospital discharge from the intensive care unit (ICU) correlated with long-term functional and cognitive performance (mean follow-up, 4 years) [18], suggesting that the effects of proinflammatory cytokines are not limited to the acute phase of sepsis.

#### Neuroinflammation

Proinflammatory cytokines enter the brain during sepsis by several mechanisms. LPS can activate TLRs through areas that lack BBB, such as the choroid plexus, circumventricular organ, and leptomeninges [19]. Many cytokines enter the brain through receptor-mediated endocytosis on brain endothelial cells. Receptors for IL-1β, IL-6, and TNF-α are expressed on cerebral endothelium [20–22] and systemic IL-1β and TNF-α cause cerebral endothelial activation [23]. Activation of cytokine receptors such as IL-1 receptor (IL-1R1) and TNF receptor (TNFR) elevates cytokine levels in the brain [24, 25]. Once these inflammatory mediators enter the brain tissue, microglial cells get activated [26]. Animal [27] and human [28] studies during sepsis consistently show microglial activation without any known cerebral infection [27, 29]. Immunostaining of a marker for microglia (CD68) was higher in brain tissues of patients who died of sepsis, supporting that neuroinflammation occurs after the onset of sepsis [28, 30]. Once microglia are activated, they most likely induce brain damage during sepsis [29, 31]. Sustained microglia activation enhances the production of inflammatory cytokines and reactive oxygen species (ROS), which creates a vicious cycle of increased BBB permeability and increased neuronal apoptosis [32, 33].

#### BBB dysfunction

The BBB is a specialized multicellular structure that selectively separates the brain interstitium from the contents of the blood vessels [34, 35]. The BBB is a dynamic barrier, with active communication between cells of the BBB and the brain parenchymal cells [36]. The innermost layer of the BBB is composed of endothelial cells, and the BBB regulates the paracellular permeability of endothelial cells through junctional proteins, including tight junction (TJ) proteins [37].

Increasing evidence suggests that BBB permeability is increased during sepsis. Using contrast-enhanced MRI of the brain, Towner et al. showed increased BBB leakage at 24 h in LPS-exposed rat brains in the cerebral cortex, hippocampus, thalamus, and perirhinal cortex regions [38]. Patients with sepsis-associated encephalopathy (SAE) frequently have vasogenic edema and white matter hyperintensities on MRI, indicating BBB disruption [39, 40]. How sepsis disrupts BBB is not entirely understood, but several mechanisms have been postulated.

An important pathway that could mediate BBB dysfunction is the disruption of TJ proteins induced by sepsis. TJ-associated proteins, mainly occludin, contain a putative matrix metalloproteinase (MMP) cleavage site [41]. After CLP in rats, MMP-2 and MMP-9 were detected in cerebral microvessels, and the increase in detected BBB permeability was time-related to the increase in MMP-2 and MMP-9 activation [41]. In humans, a reduction in TJ proteins expression was detected in the brain tissue of patients who died due to sepsis, indicating BBB damage [42].

Sepsis may also disrupt the BBB through the endothelial sphingosine 1-phosphate (S1P) receptor pathway, which maintains the BBB integrity by regulating the proper localization of TJ proteins [43]. It has been shown that in patients during sepsis, serum S1P levels were dramatically decreased and inversely associated with disease severity [44]. Additionally, the upregulation of endothelial caveolin-1 causes S1P to interact with intercellular adhesion molecule 1 (ICAM-1) and increases its binding affinity for peripheral immune cells [45]. Wu et al. found that caveolin-1 facilitates T-cell trafficking into the CNS via ICAM-1-mediated signaling [46].

Research suggests that TNF-α is another important mediator of BBB dysfunction in sepsis. TNF-α induces depolymerization of actin and the generation of intercellular gaps in the endothelial cytoskeleton [47]. In cultured brain endothelial cells, both TNF-α and IL-1β increased the expression of leukocyte adhesion molecules, such as ICAM-1, resulting in increased barrier permeability as measured by transendothelial electrical resistance [48]. TNF-α also increases barrier permeability through activating protein-kinase-6 and the internalization of VE-cadherin [49]. TNF-α has been reported to play a crucial role in the development of brain edema in acute liver failure, and increased BBB permeability was associated with loss of the TJ-associated protein occludin [50].

### Ischemia/hypoperfusion

Hypoxic or ischemic changes are the most common abnormalities found on brain autopsies of patients with sepsis [51]. Decreased cerebral flow and hypercoagulability are the main factors behind the development of cerebral ischemia in patients with sepsis [52]. Not surprisingly, impairment of cerebral blood flow autoregulation is also common in patients with septic shock, especially in the presence of hypercapnia [53].

The acute dysregulated inflammatory response that is meant to eradicate the infectious agent in patients with sepsis may cause hypercoagulability and vasculopathy of cerebral blood vessels leading to cerebral ischemia. Acute vascular endothelial dysfunction is a central event in the pathogenesis of sepsis, contributing to vascular permeability and impaired autoregulation of cerebral blood flow [52, 54]. Infiltration of immune cells into the brain tissue and increased proinflammatory cytokines release activate endothelial cells, resulting in the activation of the coagulation cascade. Microthrombi may then form in blood vessels and can cause cerebral ischemia [5].

Available studies suggest that hypoperfusion and ischemia can induce white matter (WM) changes and may contribute to long-term cognitive impairments in patients with ischemic stroke. For example, microstructural changes to the WM have been reported in patients with hyperacute ischemic stroke (less than 6 h) [55]. It has also been proposed that hypoperfusion may also cause long-term cognitive impairments in stroke through the accumulation of tau pathology [56, 57]. Studies investigating the role of ischemia/hypoperfusion in sepsis-associated cognitive impairments are warranted.

### Amyloid β and tau protein accumulation

Available preclinical and clinical data indicate that sepsis-associated brain injury is associated with molecular alterations that are shared with those seen in patients with Alzheimer’s disease (AD). It is well established that neuroinflammation in age-related brain diseases, such as AD, induces accumulation of Aβ and phosphorylated tau (p-tau) [58–62]. Data from animal models show that Aβ plaques and p-tau also accumulate acutely after experimental sepsis [59, 61]. It has also been shown that 30 days after the induction of sepsis, the brains of survivor animals have increased accumulation of Aβ and p-tau and this was associated with cognitive impairment [63]. In an animal sepsis model, LPS exposure in the rat resulted in hippocampal accumulation of Aβ plaques and intracellular p-tau, accompanied by behavioral deficits attributable to the dorsal dentate gyrus [61]. In another study, it was shown that both sepsis and aging induce brain inflammation, oxidative damage, and accumulation of Aβ, as well as impaired behavioral learning and memory function [62].

Although most of the data that show that sepsis triggers the accumulation of Aβ and p-tau in the brain come from animal research, there are several pieces of evidence that support the hypothesis that sepsis also triggers Aβ and p-tau accumulation in the brain of patients. In patients with sepsis, serum levels of tau protein are higher in those who had SAE than those who did not, and tau levels are independently associated with SAE [64]. In a prospective observational study, Orhun et al. investigated the association between neuroimaging features of sepsis-induced brain dysfunction and neuroinflammation and neurodegeneration factors [65]. The authors found that brain atrophy on MRI brain was associated with an increased serum level of p-tau. Regression analysis also identified an association between C5a levels and the presence of lesions on MRI and p-tau levels and the presence of atrophy on MRI [65].

Ehler et al. showed that in postmortem brain tissue from 5 patients who died as a result of sepsis there was diffuse staining of axons for Aβ precursor protein (β-APP). The authors also found amyloid plaque in a 79-year-old patient whose medical history did not suggest cognitive impairment [40]. Rogne et al. used 18^F^-flutemetamol positron emission tomography to visualize Aβ accumulation in patients with brain abscess and found that in 3 patients out of 17 there was an accumulation of Aβ [66]. Kramer et al. also found that periodontal infection was associated with higher Aβ load in the brain of normal elderly as measured by PIB–PET scan [67]. Kantonen et al. detected strong APP immunoreactivity in autopsies of patients with COVID-19 [68]. In a more recent study, published as a preprint, Rhodes et al. found numerous Aβ deposits in the neocortex of patients under the age of 60 who died with COVID-19 [69].

Excessive phosphorylation of tau protein is the main cause of tauopathy in AD, and glycogen synthase kinase-3β (GSK3β) is one of the most important kinases of the tau protein [70]. In addition, GSK3β is a powerful regulator of inflammation. The NF-κB pathway is a key player for the proinflammatory actions of GSK3β [71–75]. GSK3β is necessary for full stimulation of the production of several pro-inflammatory cytokines, including IL-6, IL-1β, and TNFα, and inhibition of GSK3β greatly reduces the production of pro-inflammatory cytokines [73, 75]. GSK3β regulates the inflammatory response by differentially affecting the nuclear amounts of transcription factors NF-κB subunit p65 and cAMP response element-binding protein (CREB) interacting with the coactivator CREB-binding protein (CBP) [73]. Signaling of rapamycin complex 1 (mTORC1) and GSK3β converge, and the capacity of mTORC1 to regulate the inflammatory response is related to the inactivation of GSK3β [76]. In addition, GSK3 participates in modulating the downstream signaling induced by IFNγ and the JAK/STAT pathway [77].

The receptor for advanced glycation end products (RAGE) has been identified as an important link between inflammation, amyloidogenesis, and apoptosis during AD progression [78, 79]. RAGE binds to several DAMPs, including advanced glycation end products (AGEs) [80], S100 [81, 82], and DNA [83]. In patients with AD, the plasma levels of soluble RAGE (sRAGE) are lower than in normal elderly and associated with cognitive deterioration [84]. Analysis of brain tissue from patients with AD compared to individuals without dementia showed upregulation of RAGE expression in microglia and neurons in the hippocampus, entorhinal cortex, and superior frontal gyrus [85]. By employing transgenics with targeted neuronal overexpression of RAGE and mutant APP, Arancio et al. showed that RAGE is a cofactor for Aβ-induced neuronal perturbation in a model of AD-type pathology [86]. Fang et al. showed that transgenic mice expressing human mutant APP in neurons and RAGE in microglia displayed enhanced IL-1β and TNF-α production, increased infiltration of microglia and astrocytes, accumulation of Aβ, reduced acetylcholine esterase activity, and accelerated deterioration of spatial learning/memory [87].

Available data indicate that RAGE is also involved in the pathogenesis of sepsis-associated brain injury. In the CLP animal model of sepsis, the levels of RAGE and p-tau are increased in the brain [63]. In addition, injection of RAGE antibodies into the hippocampus reduces Aβ and p-tau accumulation [60]. It has also been shown that RAGE-dependent signaling pathway regulates β- and ɤ-secretase cleavage of APP to generate Aβ, at least in part through activation of GSK3β and p38 MAP kinase [88].

### Direct brain damage

Patients with sepsis may have focal lesions that cause direct damage to the brain. Sharshar et al. reported various focal lesions in 23 patients who died in the ICU from septic shock: ischemia (100%), hemorrhages (26%), hypercoagulability syndrome (9%), micro-abscesses (9%), and multifocal necrotizing leukoencephalopathy (9%) [89]. Severe sepsis can induce life-threatening coagulopathy, including disseminated intravascular coagulation (DIC) and intracerebral hemorrhage [90]. Focal brain lesions should be considered in patients with sepsis who show signs of cognitive impairment.

Other mechanisms, such as mitochondrial and neurotransmitter dysfunction, age-related changes, and iatrogenic factors may also be involved in the pathogenesis of brain damage in sepsis. For a thorough discussion of these mechanisms, please refer to previous publications by Mazeraud et al. [29], Annane et al. [91], and Manabe et al. [92].

## Neurological manifestations of sepsis-associated brain injury

### The spectrum of acute cognitive impairments in sepsis

In the acute phase, sepsis causes several clinical syndromes that are associated with cognitive impairments, including SAE, delirium, sickness behavior, and cerebral ischemia and hemorrhage [93]. For unclear reasons, acute cognitive impairments may sometimes persist for a long time even after sepsis has improved. Currently, the management of acute sepsis-associated brain injury is still focused on treating sepsis as the underlying disease, with no specific treatments to address brain dysfunction. While this is the case, it is encouraging that appropriate management of sepsis can help prevent cognitive impairments [91, 94].

#### Sepsis-associated encephalopathy

SAE is a key manifestation of sepsis, ranging from delirium to coma [29]. SAE is defined as cognitive dysfunction associated with sepsis, in the absence of infection in the central nervous system, structural brain injury, or other metabolic abnormalities [94]. Clinically, SAE is manifested by confusion, anxiety, irritability, depression, anhedonia, decreased social communication and environmental interest, and other cognitive changes, including decreased concentration and learning capacity and impaired memory [94]. SAE is a relatively common cause of altered mental status in critically ill patients admitted to the ICU, and its prevalence varies from 8 to 70% depending on the inclusion and exclusion criteria [95]. Sonneville et al. reported that 53% of 2513 patients with sepsis admitted to the ICU had SAE, which was associated with a higher mortality rate, higher use of ICU resources, and a longer hospital stay [96]. The pathophysiology of SAE is diverse, and the mechanisms by which sepsis induces cognitive dysfunction in patients with SAE are complex and may include cerebrovascular damage, BBB disruption, neuroinflammation, neurotransmitter, and mitochondrial dysfunction, oxidative stress, and severe glial (microglia and astrocytes) activation [29, 91]. For a thorough discussion of the roles of systemic inflammation, neuroinflammation, BBB dysfunction, ischemia/hypoperfusion, and Aβ and tau accumulation in sepsis-associated cognitive dysfunction, please refer to “Underlying mechanisms of sepsis-associated brain injury” section of this manuscript. For additional information on other mechanisms, such as mitochondrial and neurotransmitter dysfunction and iatrogenic factors, please refer to published reviews by Mazeraud et al. [29] and Annane et al. [91].

#### Delirium

Delirium has many shared characteristics and symptoms with the acute phase of SAE: agitation, hallucinations, reduced concentration, and alteration of the sleep–wake cycle [97]. Delirium is commonly seen in critically ill patients and is clinically diagnosed using screening tools, such as the Confusion Assessment Method for the ICU (CAM–ICU) or the Intensive Care Delirium Screening Checklist (ISDSC). Zhang et al. reported sepsis as a risk factor for delirium in ICU patients; others reported risk factors include a history of hypertension, hypoxemia, use of benzodiazepines, deep sedation, and mechanical ventilation [98].

There is still much to learn about the mechanisms underlying sepsis-associated delirium; however, the current understanding is that its pathophysiology involves a combination of neuroinflammation and disturbances in cerebral perfusion, BBB, and neurotransmission [99]. Girard et al. identified four clinical delirium phenotypes: hypoxic, septic, sedative-associated, and metabolic delirium, which were defined a priori based on clinical judgment as well as common risk factors and hypothesized mechanisms for delirium during critical illness [100]. The authors found that longer durations of each one of these phenotypes predicted worse long-term cognitive impairment. Sedative-associated delirium was the most common phenotype and predicted more profound long-term cognitive impairment [100].

#### Sickness behavior

Patients who experience infection show common symptoms of sickness, such as loss of appetite, sleepiness, withdrawal from normal social activities, fever, aching joints, and fatigue. Collectively, this clinical syndrome is described as sickness behavior, and it has been shown that physiological concentrations of proinflammatory cytokines that occur after infection affect the brain and induce these symptoms. Sickness behavior is now recognized as part of a motivational system that reorganizes the organism’s priorities to promote recovery from infection [101, 102]. Among proinflammatory cytokines, IL-6 and IL-1β have significant relationships with sickness behavior [103]. In the CLP animal model, the severity of sickness behavior correlated with sepsis severity, mortality, increased permeability of the BBB, high level of IL-6 and oxidative stress, as well as cognitive and memory impairments [104].

### Long-term cognitive impairments in sepsis

Sepsis has various long-term effects on brain function and neurological outcomes, including alteration in mood, motor function, and cognitive impairments [4–6]. More than half of sepsis survivors have long-term cognitive impairments [91], which are still inadequately characterized, but may include loss of verbal fluency, memory, and attention deficits [4–6, 91]. Although their prevalence and temporal trajectory differ depending on the cognitive instruments used [105], cognitive impairments among ICU survivors appear to be common, severe, and persistent [105]. Furthermore, sepsis is associated with an accelerated long-term decline in global cognitive function. Compared with pre-sepsis slopes, the long-term rates of cognitive decline are faster after sepsis [106]. SAE has long been considered a reversible syndrome; however, many patients with SAE experience long-term cognitive impairments [5]. Mild to moderate neurological symptoms, including memory changes, depression, anxiety, or cognitive disorders persist in 20% to 40% of these patients 1 year after hospital discharge [94]. Acute delirium in patients with critical illness is also associated with cognitive impairments that persist long after hospital discharge [6, 107].

## Imaging findings in sepsis-associated brain injury

WM changes have a direct effect on brain dysfunction and cognitive impairments [108, 109]. Patients with sepsis-associated brain injury tend to have decreased volumes of cerebral and cerebellar WM [110]. Global WM changes on CT head imaging have been reported in adult cases of SAE [111], with no specific locations or patterns for these changes [39]. WM changes tend to worsen with a longer duration of septic shock and worse Glasgow Coma Scale scores [112].

Atrophy has also often been described in long-term observational studies of brain function after recovery from sepsis [113], and early brain atrophy may have long-term effects on the cognitive function of sepsis survivors [113]. Gunther et al. reported that a longer duration of sepsis-related delirium in ICU patients was independently associated with increased global brain atrophy and smaller hippocampal volume [114]. Furthermore, greater brain atrophy at 3 months was associated with worse cognitive performance at 12 months [114].

Apart from WM changes and atrophy, other types of pathological intracranial findings may also be present in patients with sepsis, including hemorrhages, micro abscess formation, central pontine myelinolysis, multifocal necrotizing leukoencephalopathy, and ischemic changes [115].

Although it appears that sepsis can cause widespread damage to the brain, specific anatomical locations such as the medial temporal and frontal cortex may correlate with a specific cognitive domain that has been affected in patients with sepsis [92]. Neuroimaging studies show reduced volumes of the hippocampus, amygdala, and cortex during hospitalization due to sepsis [110]. In post-mortem brain tissues from patients who died from sepsis, Sharshar et al. found that apoptotic neurons were found in the amygdala, as well as in the hypothalamus and medulla [89, 116].

New imaging modalities, such as magnetic resonance spectroscopy, diffusion tensor imaging, and positron emission tomography are promising options for early detection of sepsis-associated brain changes [39, 112, 117].

## Potential therapeutic strategies for acute and long-term cognitive impairments associated with sepsis

Based on the underlying mechanisms of sepsis-associated brain injury, we propose the following brain-directed therapeutic strategies in the acute and late phases of sepsis to help prevent or ameliorate cognitive impairments (Table 1).

### Acute phase therapeutic strategies

#### Antibiotics

Appropriate use of antimicrobial drugs is critical for the treatment of bacterial sepsis. Fluoroquinolones (e.g., ciprofloxacin, moxifloxacin), sulfonamide, rifampicin (i.e., rifampin), metronidazole, and chloramphenicol readily enter the brain, regardless of the BBB state. In contrast, more hydrophilic and larger drugs, such as vancomycin and members of the β-lactam class of antibiotics, cannot enter the brain, unless the meninges are inflamed [5]. Rifampicin should be noted in particular, because it inhibits Aβ1–40 protein aggregation and neurotoxicity in vitro [118], attenuates the inhibition of autophagosome formation and suppresses the accumulation of Aβ1–42 in vivo [119]. Furthermore, in a mouse model of LPS-induced sepsis, rifampicin protected hippocampal neurons and ameliorated cognitive impairments [119]. Iizuka et al. retrospectively reviewed FDG–PET findings of elderly patients with mycobacterium infection who were treated with rifampicin and did not have dementia at the start of treatment to examine the preventive effects of rifampicin on the progression to AD [120]. The authors found that rifampicin therapy (450 mg/day for at least 12 months) before the onset of dementia improved or stabilized AD-type hypometabolism and made metabolic decline milder in long-term follow-up after completion of therapy. Multiple regression analysis revealed that rifampicin dose and treatment duration significantly influenced FDG uptake in the posterior cingulate cortex [120].

Thus, we propose that the use of rifampicin for the acute treatment of sepsis may have the potential to prevent long-term cognitive dysfunction. However, further research is needed to test this hypothesis. Rifampicin was initially approved for the treatment of tuberculosis. Because of its low toxicity, broad-spectrum activity, and good bioavailability, rifampicin is now commonly used as part of a combination antimicrobial therapy for various infections caused by bacterial infections other than mycobacteriosis (such as acute bacterial meningitis, infective endocarditis and bacteremia, pneumonia, and biofilm-related infections) [121]. Further studies to explore the effects of rifampicin on cognitive function in sepsis should be considered. Any benefits will need to be balanced against the known complications of rifampicin use and its toxicity, including the risk for adverse drug–drug interactions and the emergence of rifampicin resistance during treatment [121].

#### Targeting proinflammatory cytokines

Proinflammatory cytokines, such as IL-6 and IL-1, are important in the early phase of systemic inflammation [4] and have been associated with cognitive impairments in sepsis [103]. Another type of brain injury caused by severe inflammation is chimeric antigen receptor (CAR)-T cell-related encephalopathy syndrome (CRES), a treatment-related complication that may occur in patients with cancer after receiving genetically modified T cells. Cytokine release syndrome (CRS) and its associated CRES neurotoxicity are caused by the release of IL-1 and IL-6, among other cytokines [122]. To suppress this inflammation, both IL-6 and IL-1 are considered viable therapeutic targets, and monoclonal antibodies (mAbs) are already established for this purpose (anakinra against IL-1R, siltuximab against IL-6, and tocilizumab and sarilumab against IL-6R). Targeting IL-1 abolished both CRS and neurotoxicity, whereas targeting IL-6 has failed to suppress delayed lethal neurotoxicity in humanized mice models [123]. In a clinical trial, anakinra was associated with significant improvement in the survival of patients with sepsis [124]. However, cognitive impairments were not assessed in this trial. Given the known role of IL-1 in sepsis, the use of mAbs against IL-1 may represent a new promising approach to suppress neuroinflammation in patients with sepsis and prevent long-term cognitive impairments, but further studies are required. TNF-α is another promising target in sepsis. In a clinical trial, TNF-α blockade therapy in patients with sepsis significantly reduced mortality 3 days after infusion, and at 28 days following treatment, a trend toward reduced mortality continued, but the difference in mortality was not significant. Neurologic outcomes were not included as part of this trial [125].

#### Prevention of hypovolemia and cerebral ischemia

Fluid replacement in sepsis is necessary to treat hypotension and hypovolemia and to prevent ischemic changes that may occur. Several studies have been conducted to determine what is the best fluid replacement strategy for sepsis. As of today, fluid replacement with crystalloid solutions is recommended [126, 127], whereas the use of colloid solutions is discouraged [128–130]. Further studies are needed to determine the efficacy of albumin-containing fluid replacement therapy [127]. Adequate fluid replacement could help prevent ischemia/hypoxia, subsequently preventing future cognitive impairments in patients with sepsis.

As noted earlier, sepsis induces hypoperfusion in the brain in a similar fashion to ischemic stroke. MRI and CT imaging studies showed that patients with SAE have similar imaging characteristics to those found in patients with stroke [39]. If neuroprotective agents (e.g., PSD-95 inhibitors [131, 132]) currently being investigated in acute ischemic stroke are proven to be beneficial, their use for early ischemic changes in patients with sepsis may help prevent long-term cognitive sequelae.

### Long-term therapeutic strategies

#### Targeting neuroinflammation

Neuroinflammation starts acutely after sepsis and persists for months [133]. Olivieri et al. showed that brain inflammation persists up to 3 months after sepsis in rats and is associated with increased Aβ level, activated microglia, and long-term cognitive impairment [133]. Microglia is the main inflammatory cell that gets activated in sepsis [26] in animals [27] and humans [28]. Thus, modulating activated microglia may be a promising approach to prevent chronic neuroinflammation and long-term cognitive impairment associated with sepsis. Recent research revealed that IL-17A/IL-17R might serve as a checkpoint in microglia-mediated neuroinflammation [134, 135]. In the CLP animal model, neutralizing anti-IL-17A or anti-IL-17R antibodies mitigated neuroinflammation and microglia activation, and alleviated cognitive dysfunction [136]. In another research using a traditional medicinal herb that is widely used for treating inflammatory conditions in South‑East Asia [137], it was shown that attractylone treatment attenuates sepsis-associated encephalopathy and cognitive dysfunction by inhibiting microglial activation and neuroinflammation [138]. Despite these encouraging data, targeting microglial activation might have drawbacks, as microglia also have neuroprotective effects [139], and inhibiting their function might have deleterious effects. More research is needed to examine targeting microglia as a therapeutic approach to help alleviate cognitive impairments associated with sepsis.

#### Targeting BBB dysfunction

BBB breakdown is an early biomarker of cognitive dysfunction in humans [140]. In patients with sepsis and SAE, vasogenic edema and WM hyperintensities on MRI are found frequently, indicating BBB disruption [39, 40]. Brain tissues of patients who died due to sepsis show a reduction of TJ proteins, indicating BBB damage [42]. Sepsis may disrupt the BBB through the S1P pathway, which maintains BBB integrity [43, 141]. Serum-S1P levels are dramatically decreased and inversely associated with disease severity in patients with sepsis [44] and in LPS-induced SAE in mice [142]. Fingolimod (FTY720) is an S1P modulator that is approved for the treatment of relapsing–remitting multiple sclerosis [143]. Fingolimod was also shown to modulate multiple neuroinflammatory markers and to decrease Aβ plaque density in a mouse model of AD [144]. Fingolimod and other S1P modulators have also shown promising results in modulating neuroinflammation [145] and BBB integrity [146] after ischemic stroke. Thus, targeting BBB disruption through modulation of the S1P pathway may be a promising direction to alleviate sepsis-induced BBB disruption and cognitive impairment, but more research is needed to test this approach.

#### Targeting amyloid β and tau phosphorylation

Growing evidence suggests that targeting Aβ oligomers (soluble aggregates of Aβ) and plaques (insoluble extracellular aggregates of fibrillar Aβ) with selective mAbs (e.g., aducanumab) can slow disease progression in patients with AD [58, 147]. Available data from animal [59, 61–63] and human research [64–69] suggest that similar to AD, Aβ and p-tau accumulation may be involved in long-term cognitive dysfunction associated with sepsis. Thus, targeting Aβ and p-tau with mAbs may also be a promising new approach to prevent or treat long-term cognitive impairments in patients with sepsis, but more research to test this hypothesis is warranted.

#### Targeting GSK3β

Excessive phosphorylation of tau is the main cause of tauopathy. GSK3β is one of the most important kinases of the tau protein [70] and a powerful regulator of inflammation [71]. This makes GSK3β another potential target for sepsis-associated brain injury. Lithium is a known inhibitor of GSK3β that has been mainly used to treat bipolar disorders and major depression. In a meta-analysis of randomized placebo-controlled trials investigating lithium as a treatment for mild cognitive impairment and AD dementia, it was suggested that lithium might have beneficial effects on cognitive performance [148, 149]. Taken together, these data and the known underlying mechanisms of sepsis-related cognitive impairments suggest that lithium might have the potential to ameliorate long-term cognitive impairments in sepsis survivors.

#### Targeting RAGE

RAGE is upregulated in microglia and neurons in the brains of patients with AD [85], and it has been identified as an important link between inflammation, amyloidogenesis, and apoptosis during AD progression [78, 79]. In animal models of sepsis, the levels of RAGE and p-tau are increased in the rat brain [63], and the injection of RAGE antibodies into the hippocampus reduces Aβ and p-tau accumulation [60]. High mobility group box 1 (HMGB1) plays an important role in inflammation and operates mainly through RAGE and TLR4 receptors [150]. In sepsis survivors, serum HMGB1 levels remain elevated at the time of hospital discharge [151], and administration of neutralizing anti-HMGB1 antibody beginning at 1 week [152] after the onset of sepsis in animals provided significant protection against cognitive impairment [152]. These data suggest that targeting RAGE directly or through HMGB1 might be a promising approach to regulate inflammation and decrease cognitive impairment in sepsis; however, more research is needed to further confirm the findings of these preliminary studies.

For discussion of other therapeutic strategies that were not mentioned in this article, please refer to Barichello et al. [4], Jarczak et al.[153], and Slikke et al. [154].

## Conclusions

In this review, we have described the spectrum of cognitive brain injuries associated with sepsis. Although the diverse molecular mechanisms underlying brain damage in sepsis are intertwined and have not been fully characterized, it is evident that local and systemic inflammation plays an important role in both the short and long term. Our proposed strategies here are meant to bring new mechanism-based directions for future basic and clinical research aimed at preventing or ameliorating acute and long-term cognitive impairments in patients with sepsis. New imaging modalities are also needed for clinical detection and characterization of sepsis-associated brain injuries.

## Data Availability

Not applicable.

## Abbreviations

Aβ: Amyloid-beta
AD: Alzheimer’s disease
BBB: Blood–brain barrier
CRES: CAR-T-related encephalopathy syndrome
CRS: Cytokine release syndrome
GSK3β: Glycogen synthase kinase-3 beta
ICU: Intensive care unit
IL: Interleukin
LPS: Lipopolysaccharide
MRI: Magnetic resonance imaging
SAE: Sepsis-associated encephalopathy
TJ: Tight junction
WM: White matter
